# Association between Metabolic Syndrome and professional category: a cross-sectional study with Nursing professionals^*^

**DOI:** 10.1590/1518-8345.5758.3529

**Published:** 2022-07-08

**Authors:** Amália Ivine Costa Santana, Magno Conceição das Merces, Argemiro D’Oliveira

**Affiliations:** 1 Universidade Federal da Bahia, Salvador, BA, Brasil.; 2 Universidade do Estado da Bahia, Departamento de Ciências da Vida, Salvador, BA, Brasil.

**Keywords:** Metabolic Syndrome, Licensed Practical Nurses, Nurses, Primary Health Care, Epidemiologic Studies, Occupational Health, Síndrome Metabólica, Técnicos de Enfermagem, Enfermeiras e Enfermeiros, Atenção Primária à Saúde, Estudos Epidemiológicos, Saúde do Trabalhador, Síndrome Metabólico, Enfermeros no Diplomados, Enfermeras y Enfermeros, Atención Primaria de Salud, Estudios Epidemiológicos, Salud Laboral

## Abstract

**Objective:**

to evaluate the association of Metabolic Syndrome and its components among Primary Health Care Nursing professionals in the state of Bahia, Brazil, according to professional category.

**Method:**

a cross-sectional, population-based and multicenter study conducted with 1,125 Nursing professionals. The independent variable was the professional category, dichotomized into technical and higher education levels. The outcome was Metabolic Syndrome following criteria from the National Cholesterol Education Program Adult Treatment Panel III based on anthropometric measurements and blood samples. The statistical analysis was performed by calculating the prevalence ratios and using Pearson’s Chi-square test.

**Results:**

the prevalence of Metabolic Syndrome was higher in the technical level category (PR=1.64; CI=1.29 - 2.06; p≤0.01). When compared to Nurses, these professionals were older, had lower incomes, worked more on duty and performed less physical activity on a regular basis. Among the Nurses, the most prevalent component was altered cholesterol (40.5%) and among the Nursing Technicians/Assistants, it was abdominal obesity (47.3%).

**Conclusion:**

the association between the Nursing category and Metabolic Syndrome was evident, with higher occurrence among technical level professionals.

## Resumo

**Objetivo:**

avaliar a associação da Síndrome Metabólica e dos seus componentes entre os profissionais de Enfermagem da Atenção Primária à Saúde do estado da Bahia, Brasil, segundo a categoria profissional.

**Método:**

estudo transversal, populacional e multicêntrico conduzido com 1.125 profissionais de Enfermagem. A variável independente foi a categoria profissional, dicotomizada em nível técnico e superior de ensino. O desfecho foi a Síndrome Metabólica seguindo os critérios da *National Cholesterol Education Program Adult Treatment Panel III*, a partir de aferições antropométricas e de amostras sanguíneas. A análise estatística deu-se pelo cálculo das razões de prevalência e pelo Teste Qui-quadrado de Pearson.

**Resultados:**

a prevalência da Síndrome Metabólica foi maior na categoria de nível técnico (RP=1,64; IC 1,29 - 2,06; p≤0,01). Estes profissionais quando comparados com os Enfermeiros eram mais velhos, tinham renda inferior, trabalhavam mais em regime de plantão e realizavam menos atividades físicas de maneira regular. Entre Enfermeiros, o componente mais prevalente foi o colesterol alterado (40,5%); entre Técnicos de Enfermagem/Auxiliares, a obesidade abdominal (47,3%).

**Conclusão:**

ficou evidente a associação entre categoria de Enfermagem e Síndrome Metabólica, cuja ocorrência foi maior entre profissionais de nível técnico.

## Resumen

**Objetivo:**

evaluar la asociación que hay entre el Síndrome Metabólico y sus componentes y los profesionales de Enfermería de la Atención Primaria de Salud en el estado de Bahía, Brasil, según la categoría profesional.

**Método:**

estudio transversal, de base poblacional y multicéntrico realizado con 1.125 profesionales de Enfermería. La variable independiente fue la categoría profesional, dicotomizada en niveles de educación técnica y superior. El resultado fue Síndrome Metabólico siguiendo los criterios del *National Cholesterol Education Program Adult Treatment Panel III* a partir de mediciones antropométricas y muestras de sangre. El análisis estadístico se realizó calculando las razones de prevalencia y utilizando la prueba de Chi-cuadrado de Pearson.

**Resultados:**

la prevalencia de Síndrome Metabólico fue mayor en la categoría de nivel técnico (RP=1,64; IC 1,29 - 2,06; p≤0,01). Dichos profesionales tenían mayor edad, menor ingreso, más guardias y realizaban menos actividad física de forma regular que los Enfermeros. En los Enfermeros, el componente más prevalente fue el colesterol alterado (40,5%); en los Técnicos en Enfermería/Auxiliares fue la obesidad abdominal (47,3%).

**Conclusión:**

fue evidente la asociación entre la categoría de Enfermería y el Síndrome Metabólico, fue mayor en los profesionales de nivel técnico.

**Highlights:**

(1) The article presents the prevalence of Metabolic Syndrome among Nursing professionals.

(2) It evidences occurrence of the problem by professional category.

(3) It concludes that there are exposure factors at work for the occurrence of the syndrome.

## Introduction

Metabolic Syndrome (MS) is a clinical condition that represent a relevant risk factor for the development of cardiovascular diseases, significantly contributing to the maintenance of the morbidity and mortality statistics in Brazil and in the world^1^. Recent studies show MS prevalence values from 8.9% to 44.0% in the Brazilian adult population, presenting an association with biological variables, lifestyle and work^2^ ^-^ ^3^.

For being a syndrome, it consists of risk factors that reflect alteration of the homeostasis of different organic systems, whose indicators are used as criteria, namely: high blood pressure, reduction in the high-density cholesterol levels, increase in the serum glucose and triglyceride levels, and visceral fat accumulation^4^. It is an inflammatory organic state, characterized by insulin resistance and, consequently, it presents a complex triad, namely: atherosclerosis; pro-inflammatory cell catharsis [emphasis on Interleukin-1 (IL-1), Interleukin-6 (IL-6), Tumor Necrosis Factor-α (TNF-α) and Ultrasensitive C-Reactive Protein (us-CRP)] and the MS components themselves^5^. Despite the cardiovascular risk already well established in the literature, these changes impose several deleterious health outcomes, such as neoplasms^6^ and increased mortality due to any cause^7^.

It should be noted that MS reflects a consequence of lifestyle, whose main conditioning factor is work, as it determines the individuals’ ways of being and living^8^. In this aspect, no studies were identified in the literature with the purpose of investigating the prevalence of MS among Nursing professionals, taking into account the occupation variable. However, it becomes necessary to discuss the health status of these professionals, since they provide an essential service in all health institutions in the world^9^. In Brazil, the Nursing profession represents more than half of the collective of human health resources and has the peculiarity of being divided into categories that presuppose different training levels and, therefore, exposure to different occupational risk factors^10^.

Despite what was proposed for the Nursing work in the National Primary Care Policy^11^, in Primary Health Care (PHC), the profession presents characteristics of both specialized and craft work. It is based on the Taylorist-Fordist way of organizing work with an emphasis on doing, on the execution of routines and flowcharts^12^. The division of tasks is striking, legitimized by the instituted hierarchy and by care fragmentation. On the one hand, we have technical level professionals who do not have the autonomy to participate in the decision-making processes that will determine the dynamics of their work and, on the other, higher education professionals who perform managerial and care activities whose perspective is to obey the protocols established, eradicating the need for production from skilled work^13^.

In this sense, organization of the work in PHC exposes Nursing workers to a situation of increasing demands with an ever decreasing degree of autonomy. These issues, associated with the current precariousness of work and employment, configure factors that can lead to psychological and physical illness in these workers, whose set of diseases includes MS^14^ ^-^ ^15^. 

Considering that the work performed by Nursing professionals in PHC can lead to the development of metabolic disorders, whose repercussions are echoed in the morbidity and mortality rates due to chronic non-communicable diseases, this study assumes scientific relevance, as it recognizes the productive sphere as an important element in the social dimension of life, presupposing an ethical dimension in the scope of Workers’ Health when acknowledging well-being at work as a right. 

The study hypothesis assumes that the occurrence of MS differs across the Nursing categories, being higher among technical level professionals. Therefore, due to the importance of the theme in question and to the fact that no papers of this nature were identified at the national level, the objective of the current research was to evaluate the association of MS and its components among PHC Nursing professionals in the state of Bahia, Brazil, according to professional category.

## Method

### Study design

This is a cross-sectional, population-based and multicenter study guided by the recommendations of the *Strengthening the Reporting of Observational Studies in Epidemiology* (STROBE) initiative^16^. Considering the territorial dimensions of the study scenario, the multicenter design allowed including individuals from the most diverse population contexts and, consequently, obtaining a broader overview of the outcome investigated, producing reliable findings to better translate the existing reality^17^. 

### Data collection locus

The study scenario was the state of Bahia (BA), Brazil, which, according to data from the Brazilian Institute of Geography and Statistics^18^, comprises 417 municipalities, organized into 7 mesoregions. For the purposes of equiprobabilistic calculation, these were stratified by clusters, whose unit was represented by the municipal unit. Through a simple random sample, 10% of the clusters of each stratum were drawn, making a total of 43 municipalities, using Microsoft Office Excel, version 2010.

### Period

The data were collected by visiting the Health Units in which the selected professionals, worked between 2017 and 2018. Prior training was offered to the interviewers to standardize the behaviors.

### Population

The study population included a representative sample of PHC Nursing professionals whose number was 9,955 workers allocated to Primary Health Care Units in the reference year: 2016^19^.

### Selection criteria

All PHC Nursing professionals over the age of 18 years old, of both genders and in full exercise of their work activities, were considered eligible and were, even, willing to undergo the collection of blood material and anthropometric measurements. The exclusion criteria were as follows: working in PHC for less than 6 months, not providing direct care to patients, or being distanced from their usual activities due to medical leave or vacations. Professionals who were pregnant, diagnosed with Burnout syndrome, depression or anxiety prior to admission to the service were also excluded, as well as patients with liver cirrhosis and those who were dependent on illicit drugs or alcohol^14^ ^,^ ^20^.

### Participants

All 1,195 Nursing professionals who worked in the PHC units of the 43 municipalities selected were considered eligible^14^ ^,^ ^20^.

Sample size calculation was based on a pilot study with a similar population. MS prevalence values were used for the exposed and non-exposed groups, of 20.0% and 33.3%, respectively. Considering an α error of 0.05, 90% power and a 1:1 ratio, and using the calculation formula for cross-sectional studies^21^, a sample of 464 professionals was obtained.

Due to the study design, the sample value was doubled, obtaining an n of 928, adding an additional 20% for possible losses and refusals, with a final sample of 1,114 Nursing professionals representing PHC in the state of Bahia, Brazil^14^ ^,^ ^20^.

### Study variables 

The exposure variable was measured using the research instrument in the block intended for the participant’s identification data. In the field entitled “profession”, the answer possibilities included the following: 1-Nurse, 2-Nursing Technician and 3-Nursing Assistant. For analysis purposes, the variable was dichotomized into “Nurse” and “Nursing Technician/Assistant”. It is noteworthy that the variable reflected the professionals’ occupation in the health unit and not necessarily their training level.

The outcome variable was MS, dichotomized into yes/no, whose diagnostic confirmation adopted the criteria of the I Brazilian Guideline for the Diagnosis and Treatment of Metabolic Syndrome^22^ and of the *National Cholesterol Education Program Adult Treatment Panel III* ^23^. Individuals who presented at least three of the five parameters considered for the diagnosis of MS were classified as “cases”: abdominal obesity (men: ≥102 centimeters-cm; women: ≥88 cm); high triglycerides (≥150 milligrams *per* deciliter-mg/dLmg/dL); low High Density Lipoprotein (HDL) cholesterol (men: <40; women: <50, or pharmacological treatment for dyslipidemia); arterial hypertension (systolic: ≥130 millimeters of mercury-mmHg and diastolic: ≥85 mmHg, or use of antihypertensive drugs); altered fasting glucose (≥110 mg/dL, or treatment for Diabetes Mellitus).

Other variables used in the analysis were the following: gender (female/male); monthly remuneration (monthly family income in minimum wages, dichotomized in up to 2 minimum wages/3 or more minimum wages); age (obtained in years old, dichotomized in up to 35 years old/36 years old or more); employment contract [type of contract in the PHC unit, dichotomized into stable (selection test or formal contract) and temporary (contracts by cooperatives, service providers or positions of trust)]; shift work (performing night work, dichotomized into yes/no); aggression at work (any episode of violence committed by a user within the PHC scope, dichotomized into yes/no); smoking (current smoking habit, yes/no answer possibilities); alcohol consumption (habitual or occasional consumption of alcoholic beverages, yes/no answer possibilities) and regular physical activities (regular and intentional weekly practice of physical exercise, yes/no answer possibilities). 

### Instruments used

Data collection involved the application of a research instrument elaborated from a literature review and containing questions regarding sociodemographic and work-related characteristics, lifestyle and human biology. The collection form was tested through a pilot study conducted with 30 Nursing professionals from a hospital. 

### Data collection

The initial approach to the professionals involved explaining the research objectives, risks and benefits, exclusion criteria considered and procedures to be adopted, such as anthropometric measurements and collection of blood material, after a 12-hour fast. In case the professionals met the criteria proposed and accepted to be part of the study population, signature of the Free and Informed Consent Form (FICF) was requested, thus guaranteeing formalization of their participation.

For the diagnosis of MS, the participants were directed to undergo the laboratory tests in a single laboratory, considered as a reference for each cluster, where blood samples were collected. Conventional enzymatic and colorimetric laboratory techniques were used to obtain the fasting blood glucose, HDL cholesterol and triglycerides serum levels^14^ ^,^ ^20^.

Abdominal obesity was verified by measuring waist circumference (WC), whose values reflect a reliable indicator of visceral adipose tissue^24^. WC was measured in a private office, safeguarding the professional’s privacy, with the individual in an anatomical position using an ISP^®^ inelastic measuring tape, in duplicate and without compressing the skin. The midpoint of the distance between the lower edge of the rib cage and the iliac bone was used as a reference body landmark, following the recommendations of the Nutrition Department of the University of São Paulo, Brazil^14^ ^,^ ^20^.

Blood pressure was measured following the recommendations of the 7^th^Brazilian Guideline on Arterial Hypertension^25^, using a stethoscope (Littmann^®^) and an anaeroid sphygmomanometer (BD^®^), both previously calibrated. Two measurements were taken on the left upper limb without clothes, with the Nursing professional sitting after a 5-minute rest (bladder emptying was requested), lower limbs uncrossed, using the mean of the last two measurements, with a 5-minute interval between them^22^.

For weight measurement, the professional was asked to wear as few garments as possible and to be barefooted. A Welmy^®^ digital anthropometric scale with a maximum capacity of 200 kilograms-kg was used. Height was measured with a retractable aluminum stadiometer, measuring up to 2 meters-m with a 0.5 cm graduation. The individual was asked to remain still, standing with the back against the device, the spine erect and the head in the Frankfurt plane^26^. With the weight and height measurements, the Body Mass Index (BMI) was calculated according to the following formula: BMI=Weight(kg)/Height(m)^2^. The BMI values considered in the analysis were dichotomized into normal weight (BMI=18.5-24.99) and excess weight (BMI≥25.00)^22^. For this variable, 11 individuals who scored as low weight, that is to say, who presented BMI values≤18.49, were excluded.

### Data treatment and analysis

The statistical analysis was carried out by descriptive analysis of the absolute and relative frequencies of the variables of interest, enabling prevalence estimates of the main dependent variable, as well as of its components. The bivariate analysis was performed using the Statistical Package for the Social Sciences (SPSS) statistical program, version 20.0, in order to verify the associations of the occupation variable with MS and its components. Prevalence Ratios (PRs), their respective 95% Confidence Intervals (CIs) and p≤0.05 were calculated using Pearson’s Chi-square test, in order to assess the statistical significance of the associations.

### Ethical aspects

The study was approved by the Committee of Ethics in Research involving Human Beings of the University of the State of Bahia (*Universidade do Estado da Bahia*, UNEB), under protocol number 872,365/2014. Collection of the information was conditioned to the participant signing the FICF and to obtaining consent for data collection, from the respective Municipal Health Secretariats of all the municipalities involved. It is noteworthy that the possibility to withdraw participation in the study was ensured at any stage of the research.

## Results

The study population consisted of 1,125 Nursing professionals (94.1% response rate), most of whom were represented by the technical level category (59.6%). It was noticed that, when compared to the Nurses, the technical level professionals were older; and that, despite having more stable contracts, they had lower incomes, worked more on duty and performed fewer physical activities on a regular basis. The occurrence of MS was higher among the Nursing Technicians/Assistants for all the variables studied (Table 1). The discrepancy between the position held and the schooling level was evident, as the number of higher education professionals (45.6%) was greater than the number of Nurses (40.4%).

**Table 1:** Sociodemographic, work-related and lifestyle characteristics of the Primary Health Care Nursing professionals (n=1,125). Bahia, BA, Brazil, 2017-2018

Variables	Nurses		Nursing Technicians/ Assistants		Metabolic Syndrome		
	n* (%)	MS^†^ (%)	n* (%)	MS^†^ (%)	PR^‡^	CI^§^	p^||^
**Gender**							
Female	391 (85.9)	17.0	598 (89.3)	28.1	1.65	1.28 - 2.13	˂0.01
Male	64 (14.1)	21.9	72 (10.7)	36.1	1.65	0.94 - 2.87	0.05
**Monthly remuneration^¶^**							
Up to 2 minimum wages	86 (18.9)	17.9	437 (65.2)	29.4	1.64	1.01 - 2.66	0.02
3 or more minimum wages	369 (81.1)	17.7	233 (34.8)	28.1	1.59	1.17 - 2.15	˂0.01
**Age**							
Up to 35 years old	320 (70.3)	16.1	267 (39.9)	22.5	1.40	0.99 - 1.96	0.03
36 years old or more	135 (29.7)	21.5	403 (60.1)	33.2	1.54	1.09 - 2.19	˂0.01
**Employment contract**							
Stable	319 (70.1)	18.4	547 (81.6)	30.2	1.64	1.26 - 2.14	˂0.01
Temporary	136 (29.9)	16.2	123 (18.4)	23.8	1.48	0.89 - 2.41	0.06
**Shift work**							
Yes	90 (19.8)	23.6	141 (21.0)	31.9	1.35	0.86 - 2.11	0.08
No	365 (80.2)	16.3	529 (79.0)	28.2	1.73	1.32- 2.27	˂0.01
**Aggression at work**							
Yes	145 (31.9)	22.9	229 (34.2)	31.3	0.42	0.32- 0.53	˂0.01
No	310 (68.1)	15.3	441 (65.8)	27.8	1.82	1.34- 2.46	˂0.01
**Smoking**							
Yes	46 (10.1)	23.9	87 (13.0)	36.8	1.53	0.85 - 2.75	0.06
No	409 (89.9)	17.0	583 (87.0)	27.8	1.63	1.26-2.09	˂0.01
**Alcoholism**							
Yes	312 (68.6)	17.3	400 (59.7)	47.8	1.72	0.72 - 4.06	0.09
No	143 (31.4)	27.8	270 (40.3)	28.3	1.63	1.28 - 2.08	˂0.01
**Performing physical activities regularly**							
Yes	303 (66.6)	14.6	336 (50.1)	26.1	1.78	1.28 - 2.48	˂0.01
No	152 (33.4)	23.8	334 (49.9)	31.8	1.33	0.96 - 1.84	0.04

*n = Number of observations; ^†^MS = Metabolic Syndrome; ^‡^PR = Prevalence Ratio; ^§^CI = Confidence Interval; ^||^p = p-value; ^¶^Current minimum wage = R$ 954.00, Brazil, 2018

**Figure 1:**
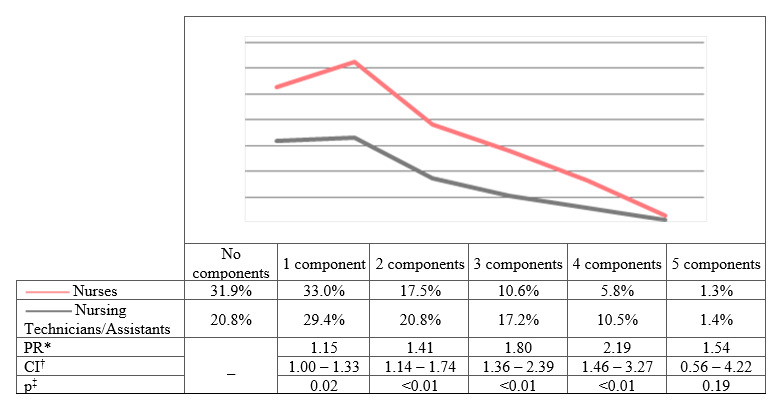
Metabolic Syndrome components according to the category of Primary Health Care Nursing professionals (n=1,125). Bahia, BA, Brazil, 2017-2018

The MS prevalence values were 29.0% among the Nursing Technicians/Assistants and 17.7% among the Nurses (PR=1.64; CI=1.29 - 2.06; p≤0.00). With regard to the MS components, in both groups, most were in the range of only 1 MS component with a decreasing occurrence trend (Figure 1).

*PR = Prevalence Ratio; ^†^CI = Confidence Interval; ^‡^p = p-value

**Figure 2:**
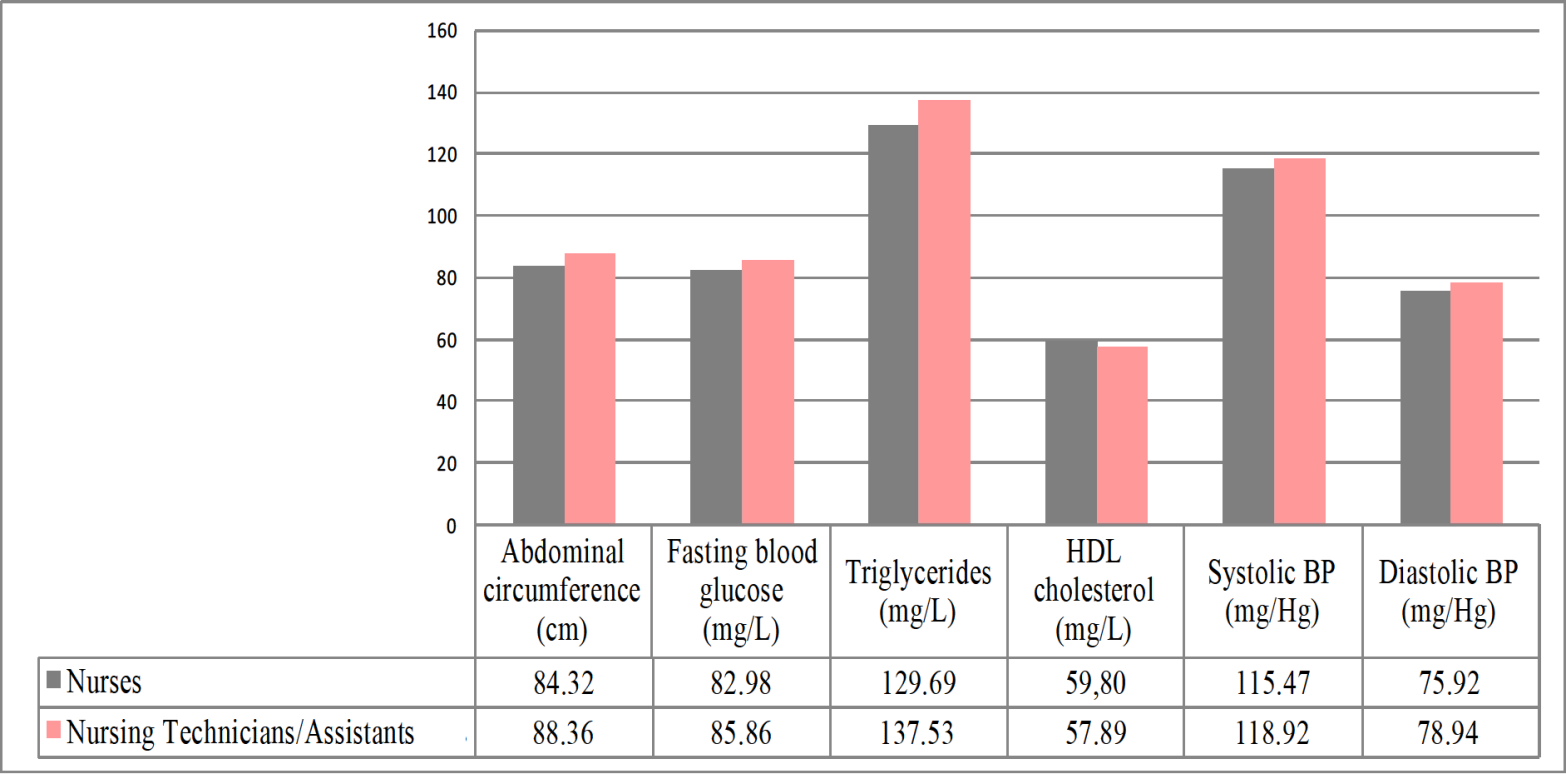
Mean values of the Metabolic Syndrome components according to the category of Primary Health Care Nursing professionals (n=1,125). Bahia, BA, Brazil, 2017-2018

Excess weight was more prevalent among Nursing Technicians/Assistants (60.1%) than among Nurses (46.3%), with a difference of 1.31 points in the mean values for BMI between the groups (PR=1.30; p<0.01; CI=1.16 - 1.46). Despite this, the Nursing Technicians/Assistants considered their quality of life as good (51.2%) and were more satisfied with their physical fitness (74.9%), when compared to Nurses. Figure 2 shows the mean values of the MS components by professional category.

Among the Nurses, the most prevalent component was altered cholesterol (40.5%) and the least prevalent was high fasting glucose (5.3%). Among the Nursing Technicians/Assistants, the highest percentage was for abdominal obesity (47.3%) and the lowest corresponded to fasting blood glucose (9.0%). The difference between the groups was evident due to the statistical significance of the associations (Table 2).

**Table 2:** Association of Metabolic Syndrome and its components with the category of Primary Health Care Nursing professionals (n=1,125). Bahia, BA, Brazil, 2017-2018

Variables	Metabolic Syndrome			p^§^	Abdominal obesity			p^§^	Hypertriglyceridemia			p^§^	Altered HDL cholesterol			p^§^	High fasting blood glucose			p^§^	Arterial hypertension			p^§^
	n* (%)	PR^†^	CI^‡^		n* (%)	PR^†^	CI^‡^		n* (%)	PR^†^	CI^‡^		n* (%)	PR^†^	CI^‡^		n* (%)	PR^†^	CI^‡^		n* (%)	PR^†^	CI^‡^	
Nurses	80 (17.7)	1.0			150 (33.0)	1.0	1.15-1.39	<0.01	130 (28.8)	1.0	1.04-1.26	<0.01	183 (40.5)	1.0	1.02-1.21	0.02	24 (5.3)	1.0	1.05 - 1.41	0.01	99 (21.8)	1.0	1.14-1.35	<0.01
Nursing Technicians/ Assistants	191 (29.0)	1.64	1.29-2.06	<0.01	317 (47.3)	1.26			241 (36.6)	1.15			306 (46.4)	1.10			59 (9.0)	1.21			217 (32.4)	1.22		

*n = Number of observations; ^†^PR = Prevalence ratio; ^‡^CI = Confidence Interval; ^§^p = p-value

## Discussion

It was noticed that the PHC Nursing workforce in the state of Bahia, Brazil, is represented by discrepant characteristics between its professional categories since, when compared to Nurses, Nursing Technicians/Assistants were older, with less healthy life habits, worked more in shifts, suffered more aggression at work and presented higher occurrence of MS, as well as of its components.

There are few studies specifically aimed at elucidating the relationship between MS among Nursing professionals in PHC, especially considering the occupation variable. This classification of Nursing by categories maintains its roots in the division of work that dates back to the beginning of the history of the profession, which, as in its beginnings, protects the division of work into essentially manual assistance and a more complex assistance work, contemplating managerial, teaching and supervision activities^27^.

In the services that are part of the Unified Health System (*Sistema Único de Saúde*, SUS) in Brazil, Nursing enjoys essentiality status, as it is impossible to think about developing its work and guaranteeing the execution of its principles without the figure of these professionals. Specifically in PHC, the service provided by Nursing professionals guarantees health care even in the absence of other professional categories in the most remote regions of the country, of continental dimensions, making us understand the importance of these professionals for the consolidation of the SUS principles^28^.

However, the international literature has already firmly established the real context of the precariousness of work for Nursing professionals, who do not even have a wage floor or social recognition in Brazil^9^. It is believed that, as it is an eminently female profession, gender issues are negatively interfering with this issue, whose struggle has been dragging on for years, without any tangible or visible achievements. A research study on MS among workers conducted from a gender perspective concluded that its occurrence differs between men and women, presenting an inverse relationship between the income and schooling variables among women^29^.

In this sense, aspects related to work are capable of interfering with the workers’ health, as they determine their way of life. A recent literature review that included research studies carried out in several countries and with various professional categories evidenced the association between MS and work-related variables, with emphasis on occupation^30^.

Occupation determines the level of remuneration, which in turn, is associated with MS. Limited resources drive the consumption of lower-cost food products, but with higher caloric contents, which favors the development of insulin resistance, hypertriglyceridemia and body weight gain. In addition to access to food, lower incomes can also interfere with the possibility of practicing physical and leisure activities, increasing the risk of MS^31^.

With a view to increasing income, it is not uncommon that Nursing professionals, especially mid-level^32^, be required to take on more than one work schedule, a finding corroborated in the current study. It is plausible to reflect on the following hypothesis: in PHC, the contractual regime for these professionals provides for 40 daytime weekly working hours, requiring night work as a form of second contract. Thus, a second employment contract determines lack of free time and, therefore, fewer physical activities and higher stress levels, with the consequent adoption of less healthy life habits, such as alcoholism, smoking and an unbalanced diet^33^. In the current study, the use of illicit drugs was reported more than twice as often by the Nursing Technicians/Assistants than by the Nurses (data not shown in the tables).

Night work as a second contract significantly modifies life habits and is capable of inducing changes in the circadian cycle, whose implications include imbalances in body metabolism. In this aspect, the literature points out that the short sleep duration imposed by shift work increases the concentration of the ghrelin hormone and, consequently, a decrease in the leptin hormone, contributing to an increase in appetite and to weight gain^34^. Other evidence has shown that poor sleep quality can activate the hypothalamic-pituitary-adrenal axis, leading to increased secretion of cortisol and catecholamines, important factors in the pathophysiology of MS^35^. The role of reduced melatonin activity as a risk factor associated with the development of MS has also been investigated^36^.

In terms of eating habits, it allows less time to prepare healthier meals, resulting in the consumption of food products with high caloric content, in addition to irregular meal times. Eating overnight can be considered as metabolically unfavorable because the body has its glucose tolerance reduced, increased gastric emptying time, and altered body temperature^37^. In addition to that, Nursing professionals who work during the night usually do not engage in regular physical activities due to the fatigue associated with the nature of their work^38^.

In the PHC context, during the workday, Nurses spend more time seated attending the office or deliberating on administrative demands. Nursing Technicians/Assistants move more during the workday and perform activities with greater energy expenditure; however, in this study, it was noticed that the latter presented higher MS prevalence values. The role of physical activity as a factor that acts in the prevention of MS is already well established in the literature, as long as it is performed regularly and intentionally^39^.

The mechanisms by which physical activity prevents the occurrence of MS include the following: (i) increase in the number of mitochondria in muscle fibers through the production of peroxisome proliferators, improving the oxidative capacity of the muscles and reducing systemic inflammation; (ii) secretion of metabolically beneficial hormones, especially irisin; (iii) reversal of insulin resistance by the muscles promoting the use of serum glucose as a substrate; (iv) reduction in hepatic lipogenesis due to increased use of fatty acids by the muscles^40^. Regular physical exercise can promote browning of the adipocytes, making them metabolically active, which ultimately culminates in thermogenesis and increased energy expenditure^41^.

It was evident that the Nursing Technicians/Assistants were more overweight, with greater waist circumference and altered BMI. Central obesity, whose most easily measured clinical counterpart is waist circumference, has shown to be a strong predictor of MS, even regardless of BMI^24^.

Diverse scientific evidence suggests that one of the main factors that can accelerate the path to obesity is insulin resistance, which, to some extent, is genetically predetermined^42^. In addition to insulin resistance, visceral adipose tissue promotes the release of non-esterified free fatty acids which, in turn, accumulate in organs such as liver and muscles, further predisposing to insulin resistance and dyslipidemia. In addition to that, the adipocytes that make up visceral fat can produce several adipokines such as leptin, resistin, TNF-α, IL-6 and angiotensin II, which can, additionally, impact on insulin resistance and pro-thrombotic and pro-inflammatory states, in addition to other cardiovascular risk factors^41^.

With regard to age, although the Nursing Technicians/Assistants were older, the study evidenced a population mainly represented by the stratum of the third decade of life, with a difference of 6 years old between the means. The relationship between age and MS remains controversial, as the wide variation in age at onset in individuals with a very similar risk profile suggests a significant interaction between genetic and environmental factors^43^. For the occurrence of obesity and pro-inflammatory state, their relationship with aging is already well established; however, despite being an important factor in the pathophysiology of MS, this variable is not unique. It is possible to assert that the work characteristics, despite individual vulnerabilities and non-modifiable risk factors, exert an impact on the occurrence of MS among Nursing professionals.

The study has limitations that deserve to be considered. Due to tis cross-sectional design, cause and effect relationships cannot be established. Due to the fact that we have only included workers in full exercise of their work activities, those who were ill, even as a result of their own work, may have been excluded from the sample. Even so, we consider that these limitations do not invalidate the findings herein presented, as the sample represents the population of PHC professionals in a large Brazilian state. In addition, the methodological rigor adopted in conducting data collection and analysis allows inferring the association between professional category and MS suggested from the results obtained.

The study contributes to knowledge as it evidences the significant prevalence of MS among Nursing professionals, affecting 1 out of 5 individuals investigated, being even higher in the technical level category. In this sense, it is assumed that these professionals seem to be more exposed to occupational exposure factors capable of culminating in the occurrence of MS. Evaluating the relationship between MS and the professional category can contribute to preventing its occurrence, representing a reduction in a significant number of cases, whose impact may be felt in the statistics of chronic non-communicable diseases.

## Conclusion

In this study, the association between professional category and MS was evident, indicating discrepant occurrence between technical level and higher education professionals. Considering the huge collective of technical level Nursing professionals in Brazil and the centrality of work in people’s lives, it becomes necessary to elucidate the possible paths for illness in this specific population, in order to reduce the negative impact of MS and guarantee assertive actions in the scope of Worker’s Health.
